# Editor's Letter-Box

**Published:** 1902-04-05

**Authors:** 


### EDITOR'S LETTER-BOX.

[Our Correspondents are reminded that prolixity is a great bar to publication, and that brevity of style and conciseness of statement greatly facilitate early insertion.]

C. R. R. Alexander writes:—I am a medical student reading for my final examination, could you advise me as to getting employment to enable me to pay my way to qualify?

[Modes of earning a living while reading for an examination must depend entirely upon individual capacity. The recent action of the Medical Council in regard to unqualified assistants has done much to limit the opportunities of medical students for finding employment during their curriculum, but it is sometimes possible to obtain some financial assistance by acting as surgery attendant to a general practitioner or even as an assistant to a chemist.]

